# 

**Published:** 1881-12

**Authors:** 


### CONTENTS:

| | PAGE. |
|---|---|
| ORIGINAL COMMUNICATIONS: | |
| Angina Pectoris. By H. R. Hopkins, M. D., | 193 |
| Acute Psoitis. By Herman Mynter, M. D., | 202 |
| On the Use of the "Radiometer" in Testing the Vision. By Lucien Howe, M. D., | 210 |
| CLINICAL REPORTS: | |
| Clinics at the Buffalo General Hospital by Professor Rochester Reported by Dr. C. C. Fredericks, | 212 |
| Pelvic Version; Report of Cases, with Remarks. By Chas. C. F. Gay, M. D., | 215 |
| Case of Forced Twin Labor at Seven Months. By Dr. L. E. Ellis, | 219 |
| TRANSLATIONS: | |
| Critical Review of Hofmeier: Upon the Value of Prophilactic Irrigation of the Uterus Immediately after Delivery. From the German by P. W. Van Peyma, M. D., | 220 |
| SELECTIONS: | |
| Note on the Relations between Syphilis and Locomotor Ataxy. By Julius Althaus M. D., M. R. C. P., | 222 |
| Acute Intestinal Obstruction Caused by Impacted Gall-Stone. By John Walters, M. B. Lond., and W. A. Berridge, M. R. C. S., Etc., | 226 |
| Pasteur's Investigations n Rabies, | 228 |
| Treatment of Chronic Prostatic Enlargement. | 230 |
| Effects of Oil of Tansy, | 232 |
| Treatment of Hernias of Long Standing, | 233 |
| Huxley's Theory of Disease, | 234 |
| EDITORIAL: | |
| A Good Lesson, | 234 |
| Examining in the Department of the Interior, | 235 |
| Will Guiteau Receive the Benefit of a Doubt? | 236 |
| A Medical Bookstore, | 238 |
| A New Journal, | 238 |
| Dr. J. Milner Fothergill on Use of Maltine, | 238 |
| REVIEWS, | 239 |

